# Where is the optimum? Predicting the variation of selection along climatic gradients and the adaptive value of plasticity. A case study on tree phenology

**DOI:** 10.1002/evl3.160

**Published:** 2020-03-10

**Authors:** Julie Gauzere, Bertrand Teuf, Hendrik Davi, Luis‐Miguel Chevin, Thomas Caignard, Bérangère Leys, Sylvain Delzon, Ophélie Ronce, Isabelle Chuine

**Affiliations:** ^1^ CEFE, CNRS, Univ Montpellier, Univ Paul Valéry Montpellier 3, EPHE IRD Montpellier France; ^2^ Institut des Sciences de l'Évolution, Université de Montpellier, CNRS, IRD EPHE Montpellier France; ^3^ Institute of Evolutionary Biology, School of Biological Sciences University of Edinburgh Edinburgh EH9 3JT United Kingdom; ^4^ INRA UR 0629 URFM F‐84914 Avignon France; ^5^ Univ. Bordeaux, INRAE, BIOGECO Bordeaux France; ^6^ Université Bourgogne Franche‐Comté UMR 6249 Chrono‐environnement 16 route de Gray, F‐25030 Besançon Cedex France; ^7^ CNRS, Biodiversity Research Center University of British Columbia Vancouver Canada

**Keywords:** Adaptive plasticity, *Abies alba*, budburst date, co‐ and counter‐gradient, elevation gradient, *Fagus sylvatica*, fitness landscape, *Quercus petraea*, selection gradient

## Abstract

Many theoretical models predict when genetic evolution and phenotypic plasticity allow adaptation to changing environmental conditions. These models generally assume stabilizing selection around some optimal phenotype. We however often ignore how optimal phenotypes change with the environment, which limit our understanding of the adaptive value of phenotypic plasticity. Here, we propose an approach based on our knowledge of the causal relationships between climate, adaptive traits, and fitness to further these questions. This approach relies on a sensitivity analysis of the process‐based model phenofit, which mathematically formalizes these causal relationships, to predict fitness landscapes and optimal budburst dates along elevation gradients in three major European tree species. Variation in the overall shape of the fitness landscape and resulting directional selection gradients were found to be mainly driven by temperature variation. The optimal budburst date was delayed with elevation, while the range of dates allowing high fitness narrowed and the maximal fitness at the optimum decreased. We also found that the plasticity of the budburst date should allow tracking the spatial variation in the optimal date, but with variable mismatch depending on the species, ranging from negligible mismatch in fir, moderate in beech, to large in oak. Phenotypic plasticity would therefore be more adaptive in fir and beech than in oak. In all species, we predicted stronger directional selection for earlier budburst date at higher elevation. The weak selection on budburst date in fir should result in the evolution of negligible genetic divergence, while beech and oak would evolve counter‐gradient variation, where genetic and environmental effects are in opposite directions. Our study suggests that theoretical models should consider how whole fitness landscapes change with the environment. The approach introduced here has the potential to be developed for other traits and species to explore how populations will adapt to climate change.

**Impact Summary**

With climate change, many species may express traits that are mismatched with their new environment. If the mismatch is too large, the population may go extinct before adapting. Mismatch can be reduced by phenotypic plasticity, through which individuals express different trait values depending on the environment, or by genetic evolution, through which individuals with genes producing traits better fitted to the environment increase in frequency in the population across generations. Unfortunately, we often have limited knowledge of which exact trait value is optimal in which environment, thereby reducing our capacity to quantify this mismatch. We here propose to take advantage of our good understanding of the causal relationships between traits, climate, and performance to predict such optimal values and how they vary across environments. We focus on the date of leaf emergence in the spring, for three emblematic Western European forest species, beech, sessile oak, and silver fir, because this trait largely affects growth, reproduction, and survival of trees. We use a model, which predictions have been extensively validated using tree distribution data and life cycle data, to predict how the optimal date of leaf emergence varies across an elevation gradient in the Pyrenean Mountains. We found that phenotypic plasticity, which results in later leafing dates at high elevation because of cooler temperature, should help all species tracking their optimal leafing date. Yet, the degree of mismatch and the consequences of this mismatch on tree performance varied both among species and with elevation. Local leafing dates were later than the optimal date in all species. While this mismatch is very small and has very little consequences for the evergreen fir, it is much larger in oak, whose performance more severely decreases at higher elevation. The approach proposed here allows calibrating models aimed at predicting populations' fate in a changing environment.

Predicting species ability to adapt to new environmental conditions requires moving forward in our understanding of the ecological bases of selection (Chevin et al. 2010; MacColl 2011; Kingsolver et al. 2012). While both genetic and plastic changes have been reported in response to contemporary climate change (see Merilä and Hendry 2014 for a review), we are still too rarely able to distinguish between adaptive versus nonadaptive responses (Ghalambor et al. 2007), and to identify the specific environmental drivers of selection (Siepielski et al. 2017). Investigating the relationship between phenotypes, fitness, and demography, that is, the fitness landscape, under variable climatic conditions is thus a priority for research on adaptation to climate change. Here, we demonstrate that, complementary to empirical estimates of selection (e.g., Chevin et al. 2015), such questions can also be investigated using process‐based models, highly trained on extensive datasets, which can predict fitness landscapes in silico. Ultimately, understanding and predicting the ability of species to adapt to climatic variation across large spatial and temporal scales will require combining such experimental and modeling approaches.

Theoretical models attempting to predict whether a species can adapt sufficiently fast to changing environmental conditions generally assume stabilizing selection around some optimal phenotype (reviewed in Kopp and Matuszewski 2014). The change in the optimum with the environment (assumed linear for simplicity), called environmental sensitivity of selection (*B*), is considered a critical parameter to predict the evolutionary trajectory of populations and their persistence (Chevin et al. 2010; Gienapp et al. 2013; Michel et al. 2014). These models generally assume that environmental change mainly affects this optimum phenotype, rather than other aspects of the fitness landscape. Under these assumptions, the mismatch between observed and optimal phenotypes in space and time is used to determine whether plasticity is adaptive or not, the strength of the directional selection, and the resulting eco‐evolutionary dynamics in a changing environment.

Plasticity can be considered adaptive if it allows tracking the spatial or temporal changes in the optimum (Ghalambor et al. 2007; Chevin et al. 2010). Quantitative assessments of the adaptive value of phenotypic plasticity are however rare (but see Vedder et al. 2013; Duputié et al. 2015; Tansey et al. 2017; Kingsolver and Buckley 2017). If plasticity alone does not allow the mean phenotype to match the optimum, we expect directional selection to locally favor genotypes with a different reaction norm, expressing phenotypes closer to the optimum. The terms co‐ and counter‐gradient variation are commonly used to describe geographical patterns where the expected genetic and environmental influences on the phenotypic cline are in the same or opposite directions, respectively (Conover and Schultz 1995; Conover et al. 2009; Ensing and Eckert 2019). Counter‐gradient variation is sometime interpreted as genetic evolution compensating for maladaptive plasticity (Crispo 2008; Grether 2005). Yet, recent theory also shows that counter‐gradient variation can adaptively evolve when environments vary both in time and space (Scheiner 2013; King and Hadfield 2019). The evolution of co‐ and counter‐gradient variation is also shaped by patterns of nonrandom gene flow and assortative mating in heterogeneous environments (Soularue and Kremer 2012, 2014). Thus, the adaptive value of plasticity cannot be inferred from the comparison of genetic and phenotypic clines across spatial environmental gradients. A better understanding of the environmental sensitivity of selection is necessary to predict both the evolution of co‐ and counter‐gradient variation and the adaptive value of phenotypic plasticity.

The environmental sensitivity of selection is however difficult to estimate. Many studies use the standardized directional selection gradient β (Lande and Arnold 1983), a measure of the strength and direction of the linear component of selection, estimated in different environments, to investigate the mechanisms and ecological factors driving selection (Caruso et al. 2017; Siepielski et al. 2017). However, variation in the selection gradient with the environment does not only depend on changes in the fitness landscape, that is, the relationship between fitness and phenotype, but also on the distribution of the phenotypes (e.g., due to past selection responses, plastic responses, or genetic drift; Chevin and Haller 2014). Conversely, in the case of perfectly adaptive plasticity, a change in the optimum across environments would not drive a change in the selection gradient. For this reason, many studies advocate assessing changes in the fitness landscape, rather than just variation in the selection gradient to reach a deeper understanding of eco‐evolutionary processes (e.g., Morrissey and Sakrejda 2013; Chevin and Haller 2014; Weis et al. 2014; Wadgymar et al. 2017). Building on a long history of selection analyses (Brodie et al. 1995; Via et al. 1995), new statistical methods have been proposed to describe how whole fitness landscapes change along continuous environmental gradients (e.g., Chevin et al. 2015; Gamelon et al. 2018), often by assuming a Gaussian shape for the fitness function.

Complementary to empirical estimates of fitness landscapes, some recent studies have used our increasing understanding of the physiological processes driving variation in phenotypes in response to environmental conditions, and of their connection with life‐history traits, to model how the optimal and realized phenotype change with the environment (Vedder et al. 2013; Gienapp et al. 2013, 2014; Weis et al. 2014; Colautti et al. 2017; Kingsolver and Buckley 2017). Building on this approach, we here show how process‐based models can be used to predict whole fitness landscapes in silico for functional traits, investigating the ecological causes of selection and the adaptive role of plasticity. The functional trait we focused on in this study is budburst date, a trait that determines the period during which temperate plant species can grow, photosynthesize, and produce their seeds (Cleland et al. 2007; Chuine 2000; Richardson et al. 2013). The exact shape of the relationship between phenology and fitness has however rarely been investigated empirically in trees (but see Bontemps et al. 2017). The process‐based model phenofit (Chuine and Beaubien 2001) is particularly relevant to address such questions as it describes the causal relationships between climate, phenology, and fitness. It also explicitly describes the major expected ecological causes of selection on budburst date in temperate trees: (1) frost damages on vegetative and reproductive organs, especially in early spring; and (2) the duration of the growing season, which determines the probability to produce viable seed. So far, the phenofit model has mainly been used to predict tree species distribution in past, current, and future climates (Morin et al. 2007; Saltre et al. 2013; Duputié et al. 2015).

Here, we make an original use of the phenofit model to investigate variation in fitness landscapes for the budburst date of three major European temperate tree species along climatic gradients. We focused our predictions on well‐studied elevation gradients in the Pyrenees Mountains, for which the phenotypic and genetic clines of the budburst date and the species performance have been characterized using long‐term in situ monitoring and common garden experiments (Vitasse et al. 2009a, 2009b). We used these datasets to validate our modeling approach at the local scale. We focused on three species that exhibit contrasting patterns of genetic variation in budburst date along elevation gradients: common beech (*Fagus sylvatica* L.), which shows a counter‐gradient pattern, sessile oak (*Quercus petraea* L.) with a co‐gradient pattern, and silver fir (*Abies alba* Mill.) for which genetic differentiation is very low (Vitasse et al. 2009a). Our method consisted of varying the budburst date in a biologically credible range and calculating the resulting fitness to obtain the relationship between fitness and budburst date for a given climate. Because this relationship is an emergent property of the model, no a priori assumption was required about the shape of the fitness function, unlike in most previous studies on similar questions (Vedder et al. 2013; Gienapp et al. 2013, 2014). We then assumed that all populations of the same species along the elevation gradient had initially the same reaction norm to climate and predicted how mismatch between mean and optimal phenotypes may result in directional selection on budburst date and lead to evolution of genetic divergence along the gradient.

We address the following questions: (1) How do the optimum and shape of the fitness landscape change along elevation gradients, and which environmental variables drive this response? (2) Is the plastic response of the budburst date always adaptive? (3) Which pattern of spatial genetic divergence would evolve starting from a single reaction norm of budburst to climate? (4) Can we explain the patterns of co‐ and counter‐gradient variation observed for these species?

## Methods

### STUDY SPECIES, REFERENCE SITES, AND CLIMATE

Common beech, sessile oak, and silver fir are emblematic species in European forests, and they co‐occur in the Pyrenees Mountains, which is characterized by a temperate oceanic climate (Vitasse et al. 2009b). While the deciduous species (beech and oak) occurs mainly at low and mid elevations, the evergreen species (fir) grows at higher elevations (above 800 m). Several populations of these species have been intensively studied along two valleys in the Pyrenees (Vitasse et al. 2009a, 2009b, 2010; Firmat et al. 2017) and were used as the reference sites for this study.

To reach broad conclusions about the spatial variation of fitness landscapes, we simulated long‐term daily meteorological data (minimum and maximum temperature, rainfall, relative humidity, global radiation, and wind speed) from 1959 to 2012 on two continuous elevation gradients, similar to the two Pyrenean valleys. The elevation effects on temperature, relative humidity, and rainfall were simulated using linear models and meteorological data acquired from 2004 to 2012 at the reference sites corresponding to the surveyed populations (see Vitasse et al. 2009b) and from the closest grid points of the SAFRAN database (see Part S1, Table S1, and Fig. S1). Global radiation and wind speed were kept constant and equal to the closest SAFRAN grid point value. We simulated local climate data every 100 m from 100 to 1700 m above sea level for beech and oak, and from 800 to 1700 m above sea level for fir. We used a principal component analysis (PCA) to describe the climatic space of the two valleys (Fig. S2).

### CALIBRATION AND VALIDATION OF PHENOFIT

**Figure 1:**
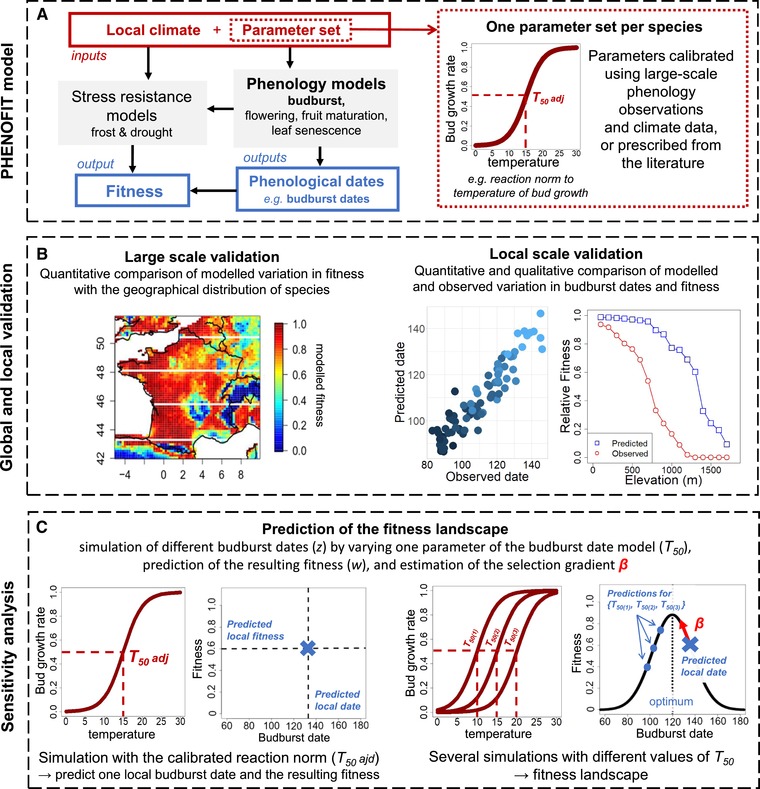
Description of the phenofit model and its calibration (A), its validation (B), and the modeling approach used to simulate the fitness landscapes (C). In the first box, the grey filled boxes represent the phenofit model. We performed a single calibration of the reaction norms describing the response of phenological/resistance traits to climate using large‐scale observations. Therefore, variation in the predicted phenological dates and reproductive success are solely due to the plasticity of the traits captured by the model, and not to potential genetic differentiation of reaction norms. We illustrate the main physiological response driving the budburst date in response to temperature. The second box illustrates a large‐scale and local‐scale predictions of phenofit that can be used to validate the species‐specific models. The third box represents the sensitivity analysis of phenofit performed to predict fitness landscapes, optimal budburst dates, phenotypic mismatch, and selection gradient for a given local climate. The variation of one parameter of a phenofit sub‐model (other parameters remaining set to the adjusted values) allows to model the relationship between budburst date (*z*) and reproductive success (*W*). Note that these schematics are for illustration purpose and do not represent the calibration or the validation outputs (results can be found in Part S2e and g).

phenofit is a process‐based model developed for temperate tree species, which mathematically formalizes causal relationships between some functional traits and environmental conditions on one hand, and between these traits and fitness (survival and reproductive success) on the other hand. In other words, the model describes explicitly how functional traits vary with environmental conditions because of their plasticity and how this variation impacts survival and reproductive success. The input of the model were the simulated daily meteorological data (see above). Functional traits simulated in the version of the model used for the study were phenological traits (leaf unfolding, flowering, fruit maturation, leaf senescence dates) as well as resistance to frost and to drought stress (Chuine and Beaubien 2001; Fig. 1A). The model assumes that the fitness of an average adult tree individual results from the synchronization between the timing of development and abiotic constraints. It calculates the annual survival probability and a proxy for the annual relative reproductive success, as the proportion of uninjured fruits that reach maturity (Part S2). The version of the model used for this study is distributed by the capsis platform (http://www7.inra.fr/capsis/).

phenofit has been previously calibrated for oak and beech using phenology observations and corresponding meteorological observations from European populations (Duputié et al. 2015). We similarly calibrated phenofit for fir using phenology observations from French populations (Part S2e; Table S2). Parameters of the frost and drought resistance sub‐models were determined using data published in the literature (Table S2). For the three species, the modeled fitness matched with a good accuracy the known distribution of the trees (data not used to calibrate the model), at the European scale for beech and oak, and French scale for fir (AUC = {0.72; 0.84; 0.88}; Fig. S3; Part S2g).

For all species, we improved the calibration of the budburst sub‐model by using phenological observations from the reference populations in the Pyrenees (monitored from 2005 to 2012), and from other French populations (http://www.gdr2968.cnrs.fr/). This calibration, across a larger range of environmental conditions than the Pyreneen ones, allowed us to maximize the robustness of the models (Table S3). We checked that these species‐specific models performed well in predicting the observed spatio‐temporal variation in budburst dates in our reference sites in the Pyrenees (R^2^ > 0.69; Fig. 1B; Fig. S3A).

Because of insufficient data to adjust population‐specific reaction norms, we used a single parameter set per species to run the simulations. We thus assess the spatial variation of fitness and budburst dates due to the spatial variation of climate only, excluding other sources of variation in selection and the potential differentiation of reaction norms. The latter simplification is however not a bad approximation of reality in our study sites and species. Indeed, Vitasse et al. (2009a) found no spatial genetic differentiation of phenology for fir in the Pyrenees. For oak and beech, spatial genetic divergence of the budburst date was significant, but explained only 2.5–3.5% of the spatial variation in budburst dates while more than 76% was due to plastic response to elevation (Vitasse et al. 2010; Firmat et al. 2017).

For this specific study, we chose as a fitness proxy the predicted arithmetic mean reproductive success of an adult tree over the 1960–2012 period because (1) the reproductive success showed much larger variation than adult survival in our simulations, as expected for long‐lived species (Fig. S4), and (2) it should reflect variation in the lifetime fitness of very long‐lived individuals, with many opportunities to reproduce and no major incidence of rare reproductive failure.

**Figure 2:**
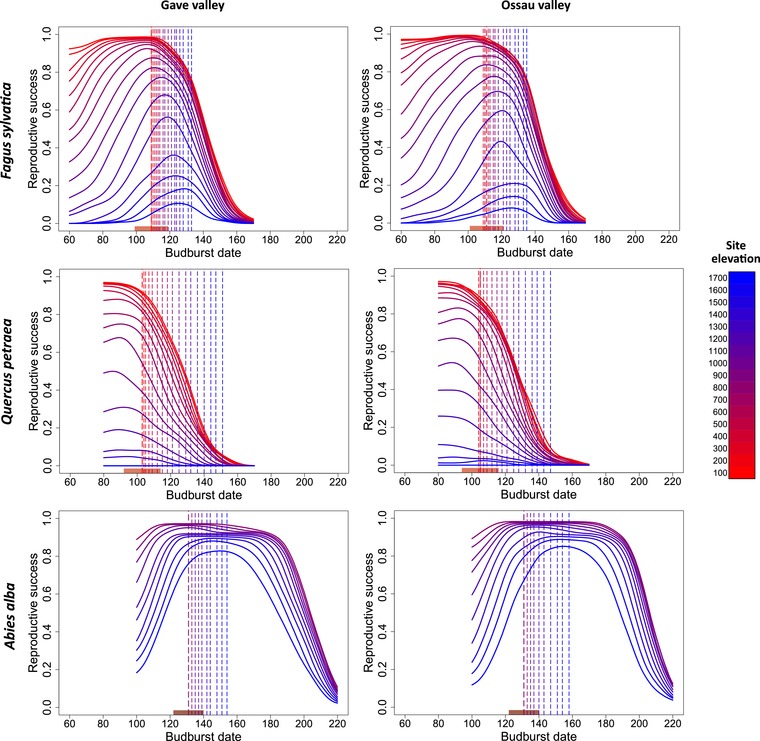
Representation of the adaptive landscapes simulated by the phenofit model at different elevations (in meters above sea level), across the two valleys, for each species. The adaptive landscapes represent the average relationship between mean population budburst date and reproductive success obtained from 100 repetitions. The dotted lines represent the predicted local budburst dates at each elevation. Depending on the species, some early and late budburst dates cannot be predicted due to specific constraints in the phenological models. Note that for oak at high elevations the simulated adaptive landscapes are null and flat. The rectangle along the *x*‐axis indicates the width of the phenotypic distribution for the lowest elevation population (as two times the phenotypic standard deviation).

We checked that the spatial variation of the predicted fitness was comparable to the observed variations of growth (for oak and beech) and acorn production (for oak) in the reference sites (e.g., no seeds produced in oak populations above 1500 m; Part S2g, Fig. S6). Note that these performance data were not used to calibrate the models. This qualitative comparison suggests that our model succeeds in capturing how climate constrains fitness for these species at a local scale (though less so at low elevations for beech) and provides another validation of our approach (Fig. 1B). For fir, we lacked fitness components data in the Pyrenees to perform such comparison.

### PREDICTING FITNESS LANDSCAPES AND SELECTION GRADIENTS

We made an original use of phenofit to study the relationship between budburst date (the phenotype *z*) and fitness (*w*), that is, the fitness function w(z), in different climates (Fig. 1C). For all species, we performed a sensitivity analysis of the model at each elevation to predict an individual fitness function. We varied one parameter of the bud growth reaction norm, previously identified as the main driver of the budburst date variation (Gauzere et al. 2019; Fig. 1A), to obtain (1) different average budburst dates over the 1960–2012 period in a biological credible range (*z*), and (2) the resulting average reproductive success over that same period w(z) for a tree with that reaction norm (Fig. 1C; Part S2h). The other parameters of the reaction norms were kept at the value calibrated for each species. Evolutionary predictions however require fitness landscapes relating mean reproductive success ($\bar{w}$) to the mean trait value ($\bar{z}$) of a population (Lande 1976). We therefore simulated populations of 1000 individuals, varying their mean budburst dates ($\bar{z}$), with $\bar{z}\in$ [60; 170] days of year (DOY) for oak and beech and $\bar{z}\in$ [100; 220] DOY for fir. For each population, individual budburst dates were drawn from a normal distribution with mean $\bar{z}$ and a standard deviation estimated from the observed budburst dates in the reference natural populations ($\sigma_{\mathrm{fir}}$ = 4.5, $\sigma_{\mathrm{beech}}$ = 5, and $\sigma_{oak}$ = 5.5; Vitasse et al. 2009b). We computed the fitness of each individual using the individual fitness function w(z), and from them the mean fitness of the population. This process was repeated 100 times per elevation to account for the uncertainty associated with population sampling.

We characterized the fitness landscapes by their maximal fitness value, optimal budburst date (i.e., the mean trait value providing the highest mean fitness) and optimal window of budburst dates (i.e., the range of budburst dates for which the fitness is higher than 95% of the maximal mean fitness). If several budburst dates produced the maximal fitness, optimal budburst date was defined as the median of these dates. We measured a proxy of the width of the fitness landscape, reflecting how slowly the fitness declines when the budburst lags away from the optimum, and thus inversely related to the strength of stabilizing selection around the optimal phenotype, as:

$$\mathrm{WFL}=\frac{\sum_{\bar{z}={\bar{z}}_{min}}^{{\bar{z}}_{max}}{\left(\bar{z}-\theta \right)}^{2}.\bar{w}\left(\bar{z}\right)}{\sum_{\bar{z}={\bar{z}}_{min}}^{{\bar{z}}_{max}}\bar{w}\left(\bar{z}\right)}$$

with $\bar{z}$ ranging from ${\bar{z}}_{min}$ = 60 to ${\bar{z}}_{max}$ = 170 for beech and oak, and from 100 to 220 for fir, and θ the optimal budburst date.

At each elevation, we then predicted the average budburst date over the 1960–2012 period (hereafter called “predicted dates”), assuming the same reaction norm to temperature at all elevations, which had been adjusted for each species. This species‐specific model of plasticity captured between 74% and 99% of the observed spatial variation in budburst dates in the reference sites (Fig. S3B). The mismatch between the predicted average budburst date and the optimal date was then used to evaluate the adaptive value of plasticity.

To predict the strength of directional selection on budburst date, we calculated variance‐scaled linear selection gradients ($\beta_{\sigma }$, defined by Lande and Arnold 1983) using the delta method to approximate it:

$$\beta_{\sigma }=\frac{{\bar{w}}_{1}-{\bar{w}}_{0}}{\Delta \bar{z}.{\bar{w}}_{0}}.\sigma$$

with ${\bar{w}}_{0}$ the mean reproductive success of a population with a mean date corresponding to the predicted budburst date (${\bar{z}}_{0}$), ${\bar{w}}_{1}$ the mean reproductive success of a population with a slightly different date (${\bar{z}}_{0}+\Delta \bar{z}$), with $\Delta \bar{z}$ = 1, and σ the phenotypic standard deviation of the trait in the population, used to produce a standardized measure of selection.

For each of these variables, variation due to sampling was represented by the interval [$\bar{x}-2\frac{\sigma_{e}}{\sqrt{100}}$; $\bar{x}+2\frac{\sigma_{e}}{\sqrt{100}}$], with $\bar{x}$ the mean and $\sigma_{e}$ the standard deviation of the variable among the 100 replicate populations.

For each species, we used linear models to estimate the proportion of variation in the optimal and predicted dates, maximal fitness, width of the fitness landscape, and directional selection gradient explained by temperature and rainfall, by using the two PCA axes defining the climatic space. In these analyses, we pooled populations from different valleys in order to infer general patterns of variation with elevation and climate (following Vitasse et al. 2009a).

### RESPONSE TO SELECTION AND EVOLUTION OF GENETIC CLINES

From the estimated variation in directional selection with elevation, we used a simple model to predict the response to selection and change in genetic values after one generation of selection. The predicted genetic cline was qualitatively compared to the species‐specific empirical clines (Vitasse et al. 2009a, b) to provide another validation of our approach. Vitasse et al. (2010) showed that beech, oak, and fir reference populations present no significant differences in the slope of the linear reaction norm of the budburst date to temperature, and that the genetic divergence of the budburst date emerges from different intercepts of these reaction norms. We similarly assumed a linear reaction norm relating budburst date (*z*) to the environment at a given elevation (ε), and that solely the intercept of that reaction norm was genetically variable within each elevation (i.e., no evolution of the slope of the reaction norm):

$${\bar{z}}_{i}={\bar{g}}_{0}+b.\varepsilon_{i}$$

with ${\bar{g}}_{0}$ the initial genetic value at elevation 0 (the intercept) and *b* the slope of the linear relationship between the trait and the environment at each elevation *i*.

We assumed that a population with an initial reaction norm, corresponding to the one calibrated using empirical observations, had colonized the elevation gradient, and that there was no gene flow among populations. While tree species are known to have large levels of gene flow, this simplification gives us an upper bound about the level of genetic differentiation that may be expected (see however Soularue and Kremer 2012). Given the Breeder's equation, and assuming that all the phenotypic variance is heritable (so as to yield an upper bound for the response to selection), the genetic value after one generation would be equal to:

$${\bar{g}}_{i}={\bar{g}}_{0}+\beta_{\sigma i}.\sigma$$

with $\beta_{\sigma i}$ the standardized selection gradient at elevation *i* and σ the phenotypic standard deviation of the trait, assumed to be the same at all elevations.

We then compared how phenotypic and genetic values varied across elevations, considering that co‐gradient variation occurred when variation in $\bar{z}$ and $\bar{g}$ with elevation were in the same direction, and counter‐gradient variation when variation in $\bar{z}$ and $\bar{g}$ with elevation were in opposite directions. When the phenotypic differentiation across gradients is mostly due to environmental effects and in a smaller extent to genetic differentiation, as observed in the case of the budburst date in our reference populations (Vitasse et al. 2010; Firmat et al. 2017), this definition of co‐ and counter‐gradient variation matches the one proposed by Conover and Schultz (1995).

## Results

### SPATIAL VARIATION OF THE FITNESS LANDSCAPES

**Figure 3:**
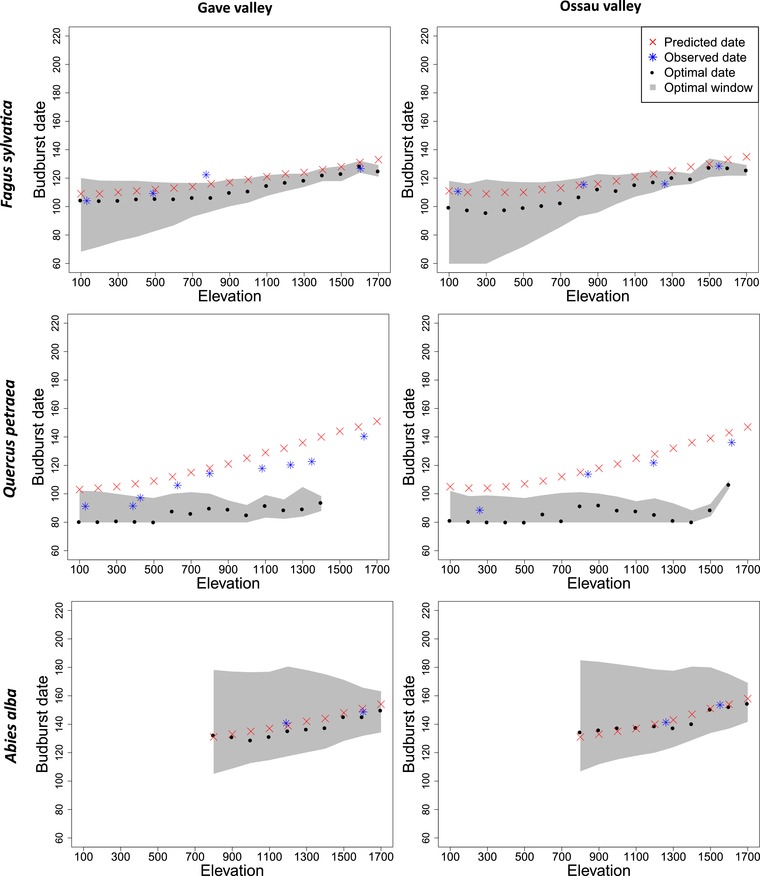
Variation in optimal and predicted budburst dates with elevation (in meters above sea level) within each valley for the three species studied. The red crosses represent the local budburst dates predicted with the phenological model calibrated for each species, that is, the response of the trait based on plasticity solely. The black points represent the budburst date providing the maximal fitness, that is, optimal date. The grey area represents the range of budburst dates covering 95% of the maximal fitness, that gives a view on the width of the adaptive peak. The blue crosses represent the observed average budburst date (2005–2012) in the reference populations. Although the predicted and observed dates are overall very similar, they are not strictly comparable as the study periods are not equivalent, and the climate along the simulated elevation gradients is not strictly identical to the climate in the reference sites.

**Figure 4:**
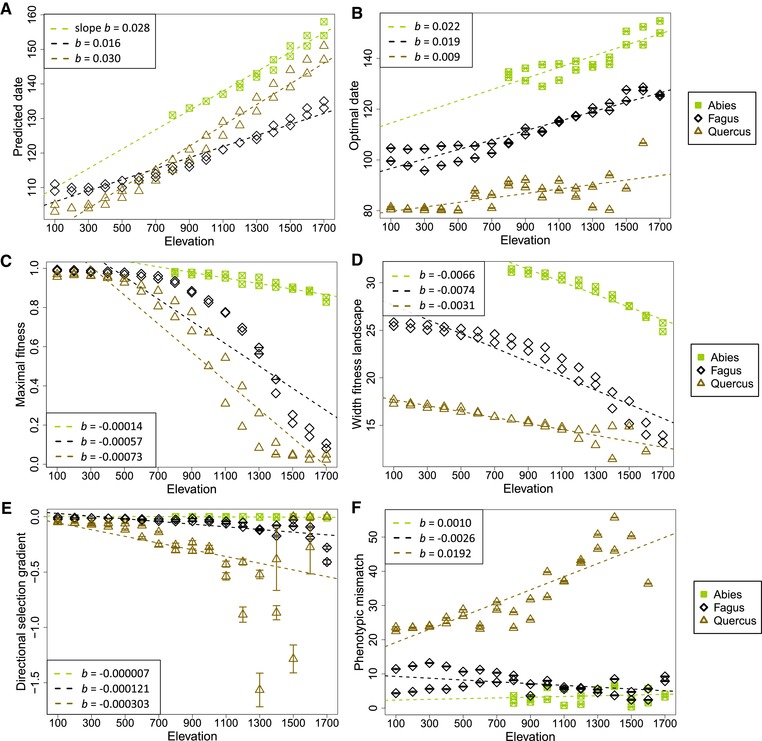
Variation of the adaptive landscapes and selective pressures acting on the budburst date with elevation for the three studied species. The unfilled points give the average parameter value and the errors bars give the uncertainty of this prediction based on 100 repetitions of the population‐level simulations. The within‐plot legends provide the species‐specific slope coefficient of linear regression with elevation. Units: predicted and optimal dates in DOY; maximal fitness a relative measure ranging from [0; 1]; width of the fitness landscape in days; standardized linear selection gradient in units of phenotypic standard deviation; absolute phenotypic mismatch in days.

For all species, the simulated fitness landscapes included a single optimum, which varied with elevation (Figs. 2 and 3). Both the optimal and the predicted budburst dates were later at higher elevations (Figs. 3, and 4A and B). At lower elevation, a larger range of budburst dates was associated with optimal fitness values. The width of this optimal window of budburst dates decreased with increasing elevation (Fig. 3). For all species, this variation in the shape of the fitness landscapes would result in an increasing strength of the within‐population stabilizing selection around the optimum with elevation (Fig. 4D). The maximal fitness value also decreased with elevation in all species (Fig. 4C). All these trends were mainly explained by the variation in temperature along the elevation gradient (Table 1).

**Table 1:** Variation of the adaptive landscapes and selective pressures on the budburst date with the climatic variables

	*F. sylvatica*				*Q. petraea*				*A. alba*			
	Axis 1		Axis 2		Axis 1		Axis 2		Axis 1		Axis 2	
Variable	Effect	η^2^	Effect	η^2^	Effect	η^2^	Effect	η^2^	Effect	η^2^	Effect	η^2^
Predicted date	−3.47	0.96	−0.92	0.01	−6.38	0.98	−1.18	0.01	−3.76	0.99	−0.43	0.00
Optimal date	−4.04	0.90	−2.07	0.03	−1.99	0.44	−0.66	0.01	−3.06	0.82	1.07	0.03
Wmax	0.12	0.84	0.05	0.02	0.16	0.92	0.03	0.01	0.02	0.78	0.01	0.14
WFL	−0.004	0.76	−0.003	0.07	−0.003	0.75	−0.002	0.07	−0.001	0.89	−0.0004	0.03
$\beta_{\sigma }$	0.03	0.55	0.01	0.003	0.08	0.22	−0.01	0.001	0.0008	0.32	0.0007	0.06
Mismatch	0.57	0.21	1.14	0.12	−4.26	0.76	−0.38	0.001	−0.70	0.21	−1.49	0.27
Average		0.74		0.04		0.55		0.01		0.64		0.10

We tested the effect of the first two axes of the PCA describing the climatic space over the elevational gradients using an ANOVA on a linear model. The table details the main effect and proportion of variance explained by each of the PCA axis, with η^2^ = SSvar/(SSvar + SSres). Axis 1 is mainly driven by the temperatures and axis 2 by the precipitations. With Wmax the maximal fitness, WFL the width of the fitness landscape and $\beta_{\sigma }$ the standardized linear selection gradient.

Additionally, our results highlighted some species‐specific patterns (Fig. 4). The variation in budburst dates with elevation due to plasticity was predicted to be higher for oak and fir (3.0 and 2.8 days/100 m, respectively) than for beech (1.6 days/100 m; Fig. 4A), consistently with the observed patterns (Fig. 3). Variation in the optimal date with elevation was more than twice greater for beech and fir (1.9 and 2.2 days/100 m, respectively) than for oak (0.9 days/100 m; Fig. 4B). The average strength of stabilizing selection, as inversely reflected by the width of the fitness landscape, and its variation across elevation were higher for the deciduous than the evergreen species (Fig. 4D). The maximal fitness also decreased more steeply with elevation for the deciduous than evergreen species (Fig. 4C). Note that because oak populations at the highest elevations had null reproductive success for all possible budburst dates (Fig. 2), no optimal trait value could be defined for them.

### PHENOTYPIC MISMATCH AND THE ROLE OF PLASTICITY

For all species, the variation in the optimal budburst dates with elevation was in the same direction as the predicted dates (Fig. 4A and B). But the extent to which the plastic response allows following changes in the optimal date varies among species. For fir, the predicted and optimal dates were almost perfectly identical, with predicted budburst dates always included in the optimal window of trait values (Fig. 3). For beech, the predicted and optimal dates were also very close, but with a constant lag, the optimum being earlier (Fig. 3). For oak, the predicted date was always far too late, especially at high elevations. The average phenotypic mismatch was higher for oak (22–56 days) than for beech (2–13 days), and almost negligible for fir (–4 to 7 days; Fig. 4F). The plasticity of the budburst date was thus found to be more adaptive for beech and fir than for oak. Using the average budburst dates observed over the recent years in the reference sites, instead of the predictions derived from the plastic model, does not modify our qualitative conclusions about the adaptive value of plasticity and strength of the phenotypic mismatch among species (Fig. 3).

### SPATIAL VARIATION IN DIRECTIONAL SELECTION

**Figure 5:**
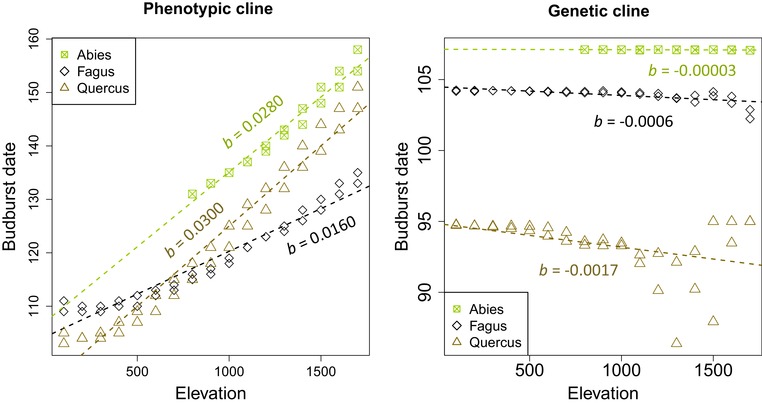
Variation of the phenotypic and genetic values for the budburst date with elevation gradients for the three studied species. Red symbols represent the predicted budburst dates based on plasticity (phenology model detailed in Part S2). Grey symbols represent the genetic values after one episode of selection, given the breeder equation and the predicted linear selection gradient. The slope coefficients (*b*) are provided over each linear regression line.

For all species, we predicted stronger directional selection toward earlier budburst date at higher elevation, and low or no directional selection gradients at low elevation (Fig. 4E). The average directional selection gradient (${\bar{\beta }}_{oak}$ = −0.34, ${\bar{\beta }}_{beech}$ = −0.07, and ${\bar{\beta }}_{fir}$ = −0.002; variance standardized measures) and its variation across elevation (oak: −0.0303/100 m; beech: −0.0121/100 m; fir: −0.0007/100 m) were higher for oak than for beech, and very low for fir (Fig. 4E). For all species, variation in the linear selection gradient was mainly driven by the temperature (22–55% of variance explained; Table 1). Using the estimated directional selection gradients, we predicted the evolution of counter‐gradient variation for oak and beech, and a negligible genetic evolution for fir after one generation of selection (Fig. 5).

## Discussion

### CAUSES AND CONSEQUENCES OF SPATIAL VARIATION IN FITNESS LANDSCAPES

The approach we developed here allowed us to predict the shape of the fitness landscape and the spatial variation in selective pressures on the budburst date of tree species. Within populations, the simulated fitness landscape, with a single optimum, reflected the trade‐off between maximizing growing season length and minimizing frost injuries on vegetative and reproductive organs. The predicted stabilizing selection on the budburst date is consistent with empirical estimates in trees (Bontemps et al. 2017). Our simulated asymmetrical fitness landscapes, with stronger decline in fitness with later dates, are also similar to recent predictions and observations on flowering dates in an annual plant (Weis et al. 2014).

We found strong variation in the shape of the fitness landscapes with elevation, in terms of optimal trait value, width of the fitness peak, and maximal fitness. In particular, the predicted delay in the optimal budburst date with elevation is consistent with the observation of later frost occurrence at higher elevation in the reference populations used in this study (Dantec et al. 2015). The sharp decline in fitness when slightly moving away from the optimal budburst date and the low maximal fitness at high elevation are consistent with empirical observations of low fecundity in the populations at the highest elevations in oak (Fig. S6). When the fitness landscape has a Gaussian shape, the directional selection gradient can be expressed as the phenotypic mismatch scaled by the width of the fitness landscape (Lande and Arnold 1983). Although predicted fitness landscapes here deviate from a Gaussian shape, variation in selection gradients with elevation was consistent with this expectation. Indeed, we predicted stronger directional selection for earlier budburst at higher elevation, because of a narrower fitness peak for all species, and a higher phenotypic mismatch for oak. The much stronger selection for earlier budburst in oak compared to the other species may be explained by a longer maturation time of its large fruits, consistent with the observation that fruit size is a critical determinant of northern range limits in trees (Morin and Chuine 2006). Contrarily to the meta‐analysis by Siepielski et al. (2017), we predicted that the variation in the shape of the fitness landscape and resulting directional selection gradients were mainly driven by the temperature, and not precipitation. Empirical estimates of selection gradient on the budburst date in the same oak populations also suggest selection for increased precocity, which increases with elevation (Caignard, Delzon and Kremer, *pers. comm*.). Comparison of predicted and empirically measured selection gradients should help validate our modeling approach (e.g., Weis et al. 2014) and, ultimately, a combination of modeling and empirical estimates should lead to greater insights about the causes of variation in selection. However, empirical estimates of selection gradients in adult trees, integrating climatic variation over years, are still very scarce.

While we predicted stronger directional selection on the budburst date at higher elevations for beech and oak, we also predicted a strong decrease of the maximal fitness with increasing elevation. This result suggests that, even if populations at high elevations evolved to their optimal budburst date, they may not be demographically viable in such environments (Chevin et al. 2010). Finally, our predictions suggest that care should be taken when applying predictions from simple theoretical models of phenotypic adaptation to specific empirical contexts. Even though basic aspects of these models were verified here (e.g., gradual shift in the optimum phenotype with the environment), we found that the tree species did not conform to the common assumption of invariable width or height of the fitness landscape along ecological gradients (Chevin et al. 2010; Gienapp et al. 2013).

### ADAPTIVE PLASTICITY AND GENETIC EVOLUTION OF PHENOLOGY

The role of plasticity in the adaptive response to environmental variation at short time and fine spatial scales is increasingly acknowledged (Baythavong 2011; Vedder et al. 2013; Merilä and Hendry 2014; Phillimore et al. 2016). Variation in mean phenotypes along environmental gradients, resulting from both phenotypic plasticity and genetic differentiation, is often assumed to reflect variation in optimal phenotypes through space (e.g., Tansey et al. 2017). Whether such an equilibrium has been reached for long‐lived tree species is however questionable (Savolainen et al. 2004). Here, instead of assuming that the observed clinal variation was optimal, we predicted the optimal phenotypes with a process‐based model, and compared them to the local phenotypes also predicted by the model. We found that plasticity would be almost perfectly adaptive in fir, and partially adaptive in the two deciduous species, although more adaptive in beech than oak, the latter exhibiting hyperplasticity (i.e., its environmental response is too high to track the optimum). This result is consistent with previous studies looking at the adaptive value of the budburst date plasticity in beech and oak at larger spatial scales (Tansey et al. 2017), and particularly with the study by Duputié et al. (2015), which relied on the same type of modeling as ours.

We used our model to predict how directional selection may shape the evolution of budburst along environmental gradients after one generation. Genetic clines in budburst date along elevation gradients, measured in common gardens, indicate counter‐gradient variation in beech, co‐gradient variation in oak, and negligible genetic differentiation in fir (Vitasse et al. 2009a; Alberto et al. 2011). Similar patterns have been reported for fir and beech across other elevation gradients (Gauzere et al. in review, Latreille et al. *pers. comm*.). Our predictions in fir and beech are consistent with these observations, which provides empirical support for the biological relevance of our approach. Interestingly, predictions for beech suggested that a pattern of counter‐gradient variation may evolve even in the absence of hyperplasticity, contrary to the expectation that counter‐gradient variation is caused by excessive plasticity (Conover and Schultz 1995). The discrepancy between simulated and measured genetic clines in oak may be explained by the fact that we do not simulate assortative mating and gene flow among populations. Indeed, nonrandom mating can generate patterns of co‐gradient variation even in the absence of divergent selection on spring phenology (Soularue and Kremer 2012).

### ADVANTAGES AND LIMITS OF THE PROCESS‐BASED MODELING APPROACH

The modeling approach developed and used in this study allows simulating fitness landscapes that are particularly difficult to estimate in natural populations. While empirical measurements are essential, such a process‐based modeling approach can be used to predict the long‐term consequences of trait variation on fitness, and explore larger temporal and spatial scales than experimental studies. This approach has the potential to explore the consequences of future environmental changes, such as climate change, on trait maladaptation and its consequences on population persistence (e.g., Phillimore et al. 2016; Kingsolver and Buckley 2017), when empirical estimates of selection in future climates generally rely on the problematic space for time paradigm. Predicting fitness landscapes from process‐based models also has the main advantage that it does not require any assumptions about the shape of selection on traits (see also Weis et al. 2014).

While this approach is based on our eco‐physiological understanding of tree performance and extensive observations from natural populations, our conclusions depend nevertheless on the accuracy of the predictions of the model used. For this reason, a thoughtful prior validation of the model predictions is necessary. Although model predictions generally agreed with observations in this study, our understanding of selection could be improved by exploring other physiological processes and life‐history components. For example, carbon uptake and allocation might also affect fitness (Delpierre et al. 2016), and were crudely modeled in the present version of phenofit. A promising prospect would be to apply the approach developed here to other process‐based models that focus on other physiological processes and fitness components (e.g., Davi et al. 2009). Strength of selection and optimal values may differ across life‐history components (e.g., juvenile stages, growth, survival, and fecundity) with complex consequences for eco‐evolutionary dynamics (Cotto et al. 2019).

We assumed a dominant effect of plasticity over genetic differentiation in driving the initial phenotypic variation among populations. This simplification was acceptable for the study species and sites (see Vitasse et al. 2010; Firmat et al. 2017). However, when genetic divergence has strong effects on phenotypic variation across sites compared to environmental effects, different reaction norms should be used for the different populations to predict patterns of local selection, which require more observational and experimental data (e.g., Chuine et al. 2000; Fournier‐Level et al. 2016). A better knowledge of natural variation in physiological responses across species distributions is currently one of the main challenges that needs to be addressed to properly integrate genetic evolution in process‐based models (Liang 2019; Benito Garzón et al. 2019).

## Conclusion

We predicted that plasticity of the budburst date in fir, oak, and beech helps them adapt to climatic gradients. However, in deciduous species, plasticity was not sufficient to perfectly track the spatial variation in the optimal date, resulting in selection for increased precocity with elevation. Most importantly, our study suggests that focusing only on changes in optimal trait values, and neglecting other changes in the shape of the fitness landscape, may be misleading about the role of plasticity and evolution in heterogeneous environments. The approach developed here could be applied to other species and other functional traits, owing to the growing panel of process‐based models developed for plants and animals that explicitly relates environment, traits, and fitness (Kearney and Porter 2004; Weis et al. 2014; Burghardt et al. 2015; Kingsolver and Buckley 2017). They could especially be used to examine the evolutionary and population dynamics underlying range limits in changing climates.

Associate Editor: S. Wright

## Supporting information


**Table S1**. Coefficient values for the equations used to simulate the elevation gradients for each valley and species.
**Table S2**. Parameter values of the PHENOFIT model used for each species.
**Table S3**. Statistical performance of the budburst model.
**Figure S1**. Location and reference of the SAFRAN reference points.
**Figure S2**. Climatic characterization of the simulated elevational gradients.
**Figure S3**. Global‐scale validation of the PHENOFIT model using observed species distribution range and AUC.
**Figure S4**. Spatio‐temporal variation in observed and predicted budburst dates for the reference sites in the Pyrenees.
**Figure S5**. Reproductive success and survival predicted by the PHENOFIT model across the reference sites in the Pyrenees.
**Figure S6**. Spatial variation in predicted fitness and observed performance of beech and oak trees in the Pyrenees (Gave valley).
Data **S1**.
