# Supplementary material for: Where is the optimum? Predicting the variation of selection along climatic gradients and the adaptive value of plasticity. A case study on tree phenology
Source: Evol Lett. 2020 Mar 10;4(2):109–23. doi: 10.1002/evl3.160 (PMC7156102; doi:10.1002/evl3.160)

Supporting Information for
Where is the optimum? Predicting the variation of selection along climatic gradients and the adaptive value of plasticity. A case study on tree phenology.

Gauzere J.[1,2], Teuf B.[1], Davi H.[3], Chevin L.M.[1], Caignard T.[4], Leys B.[1,5], Delzon S.[4], Ronce O.[2]*, Chuine I.[1]*

[1] CEFE, CNRS, Univ Montpellier, Univ Paul Valéry Montpellier 3, EPHE, IRD, Montpellier, France
[2] Institut des Sciences de l'Évolution, Université de Montpellier, CNRS, IRD, EPHE Montpellier, France
[3] INRA, UR0629 URFM, F-84914 Avignon, France
[4] INRA, University of Bordeaux, UMR BIOGECO, F-33615 Pessac, France
[5] Université Bourgogne Franche-Comté, UMR 6249 Chrono-environnement, 16 route de Gray, F-25030 Besançon Cedex, France

Contents

1. Climatic data
Part 1. Description of model used to simulate climatic data.
Table S1. Coefficient values for the equations used to simulate the elevation gradients for each valley and species.
Figure S1. Location and reference of the SAFRAN reference points.
Figure S2. Climatic characterization of the simulated elevational gradients.

2. The PHENOFIT model
Part 2. Description of the version of model used in this study.
- 2.a. Phenology models
- 2.b. Survival to stresses
- 2.c. Reproductive success
- 2.d. Input and output variables
- 2.e. Model calibration
- 2.f. Models simulations
- 2.g. Model validation
- 2.h Local sensitivity analysis

Table S2. Parameter values of the PHENOFIT model used for each species.
Table S3. Statistical performance of the budburst model.
Figure S3. Global-scale validation of the PHENOFIT model using observed species distribution range and AUC.
Figure S4. Spatio-temporal variation in observed and predicted budburst dates for the reference sites in the Pyrenees.
Figure S5. Reproductive success and survival predicted by the PHENOFIT model across the reference sites in the Pyrenees.
Figure S6. Spatial variation in predicted fitness and observed performance of beech and oak trees in the Pyrenees (Gave valley).

1. Climatic data

Since our aim was to reach broad conclusions about the spatial variation of adaptive landscapes with elevation, we generated long-term meteorological data from 1959 to 2012 on two continuous elevation gradients corresponding to the two focal valleys. To recreate realistic elevation gradients, we used temperature data recorded by local weather stations (Hobbo sensors) in the references populations along the Gave and Ossau valleys (see Vitasse *et al.* 2009b for more details). As these local sensors only recorded temperature data over the 2004-2012 period, we also used data from the SAFRAN database to simulate climatic data over a larger historic period. SAFRAN database consists in reanalyzed climatic data covering France at an 8-km resolution. We thus simulated the temperatures variables for all elevations by combining the locally estimated slopes of temperature vs elevation using the local Hobbo sensors data, and temperature data over the period 1959-2012 for a point of the SAFRAN grid at low elevation for each of the two valleys: point SAFRAN 479 for the Ossau valley (located at 576m) and point 484 for the Gave valley (located 461m; Fig. S1). We used the following equation to simulate daily minimum and maximum temperatures (T) over the 1959-2012 period:

$$T(X) = T_{SAFRAN} + (elevation_X - elevation_{SAFRAN}).\delta_T + \Delta_T \quad (1)$$

where T_{SAFRAN} is the daily temperature extracted from the SAFRAN database, Δ_T is the offset of temperature between the SAFRAN estimate and Hobbo measurements, and δ_T is the gradient of temperature with elevation estimated using Hobbo data. This implies that the variability of the simulated climate is the same at all elevations.

As the Hobbo sensors do not record precipitation and relative humidity, we only used SAFRAN data over the 1959-2012 period to estimate gradients of relative humidity and rainfall with elevation for the two valleys. As the resolution of our study sites is higher than that of SAFRAN grid, we only used two points to estimate these gradients: one grid point at low elevation (the same than for the temperature simulation), and one point at high elevation. To estimate the rainfall and relative humidity gradients, we used 15 points of the SAFRAN grid located in the two valleys studied and in the geographic extent defined by the plots studied (Fig. S1). We simulated relative humidity (*RH*) and precipitation (*Rain*) at each elevation using these equations:

$$RH(X) = (RH_{SAFRAN} + (elevation_X - elevation_{SAFRAN})).\delta_{RH} \quad (2)$$

where RH_{SAFRAN} is the daily relative humidity extracted from the SAFRAN database, δ_{RH} is the gradients of relative humidity with elevation estimated using SAFRAN data.

$$Rain(X) = Rain_{SAFRAN}.\left(1 + \frac{(elevation_X - elevation_{SAFRAN}).\delta_{Rain}}{dayNumber.mean(Rain_{SAFRAN})}\right) \quad (3)$$

where $Rain_{SAFRAN}$ is the daily precipitation extracted from the SAFRAN database, δ_{Rain} is the annual gradients of rainfall with elevation estimated using SAFRAN data, $dayNumber$ is the number of days during a year, so that the linear variation of rainfall with elevation is estimated based on annual measurement, but the simulated variable is at daily scale.

Daily wind speed and global radiation were kept constant and equal to the SAFRAN grid

point value along the elevation gradient as there were no simple ways to relate these variables to elevation.

Using these equations and the coefficient values provided in Table S1, we simulated two elevational gradients composed of 34 sites ranging from 100 to 1700 m in south-facing slopes for beech, 34 sites ranging from 100 to 1700 m in north-facing slopes for oak and 20 sites ranging from 800 to 1700 m in south-facing slopes for fir. The simulated elevation gradients were described by the first two axes of a principal component analysis using six climatic variables (Fig. S2).

Table S1. Coefficient values for the three equations used to simulate the elevation gradients for each valley and species. With δ_{TMAX}, δ_{TMIN}, Δ_{TMAX} and Δ_{TMIN} in $^\circ$C / 100 m, δ_{RH} in % / 100 m and δ_{Rain} in mm / 100 m.

Source	Valley	δ_{TMAX}	δ_{TMIN}	Δ_{TMAX}	Δ_{TMIN}	δ_{Rain}	δ_{RH}
Hobbo	Gave North	-0.4592	-0.3922	2.135	-0.8955		
Hobbo	Gave South	-0.4886	-0.3849	2.712	0.2842		
SAFRAN	Gave					30.96	0.1918
Hobbo	Ossau North	-0.5552	-0.4217	1.5291	-2.1785		
Hobbo	Ossau South	-0.4416	-0.4300	2.404	-1.052		
SAFRAN	Ossau					42.35	0.3847

Figure S1. Location and reference of the SAFRAN points (blue triangles and numbering) used to simulate the climatic gradients in this study. The location of the reference populations along the Ossau and Gave valleys are represented by black points. The x- and y-axis represent the longitude and latitude of the sites respectively, and the color scale represents the variation in elevation (m).

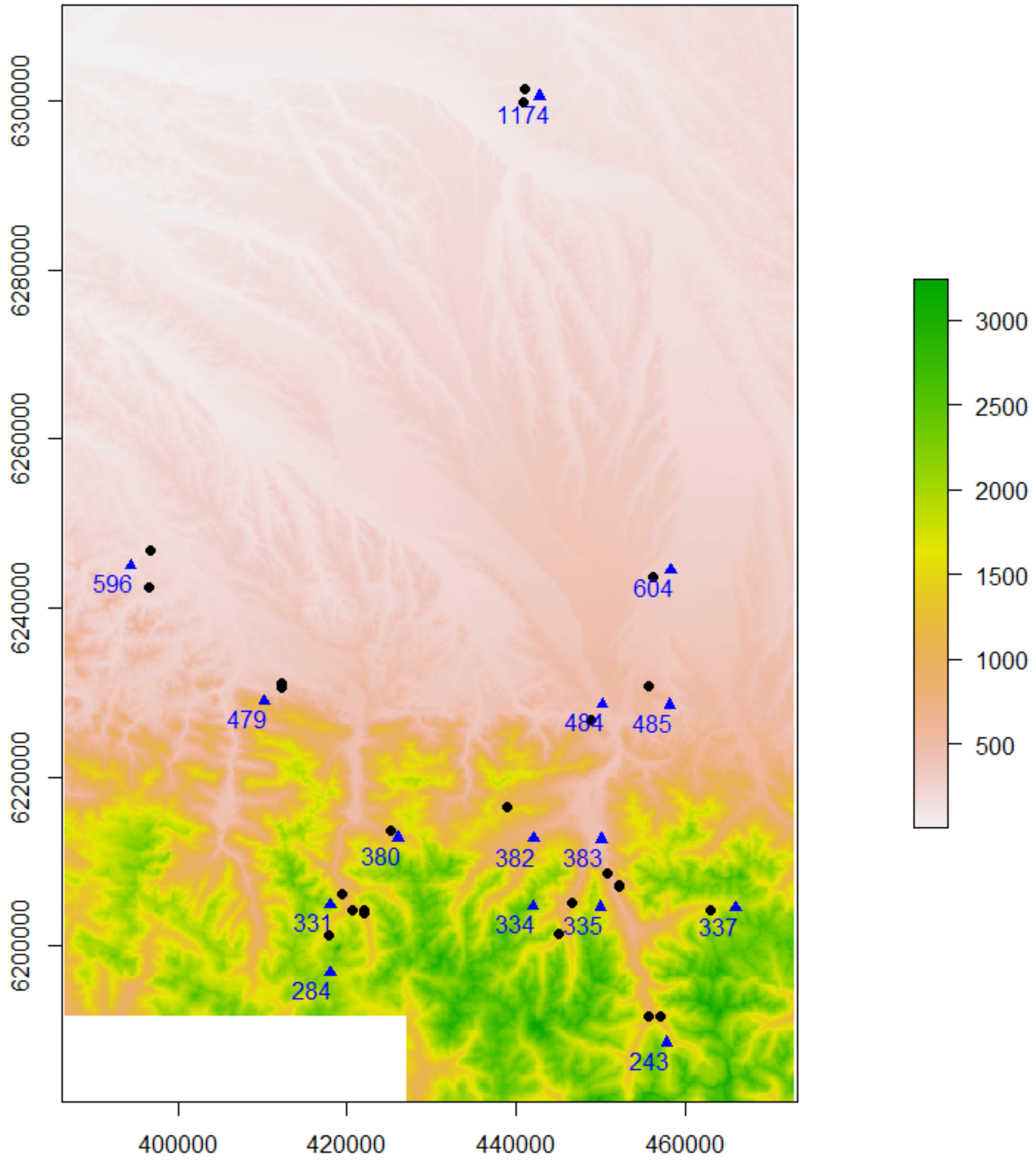

Figure S2. Climatic characterization of the simulated elevational gradients, obtained from a principal component analysis. Each elevation gradient is composed of 17 sites ranging from 100 m to 1700 m a.s.l.. These gradients reproduce climate variation in two Pyrenean valleys (Luz and Ossau). North- and south-facing gradients were simulated to match with the ecological requirements of the studied species. Six variables were used to describe the climatic space (following Duputie *et al.* 2015): *"moyTmp"*: the average temperature during the warmest month (°C), *"GDD"*: the growing day degrees above 5°C over the vegetative period (°C), *"moyTmin"*: the average minimal temperature during the coldest month (°C), *"nbChillDays"*: the number of chilling days (days), *"CumulPrepW"*: the amount of precipitations during the warmest month (in mm), *"CumulPrep"*: the total precipitations over the year (in mm).

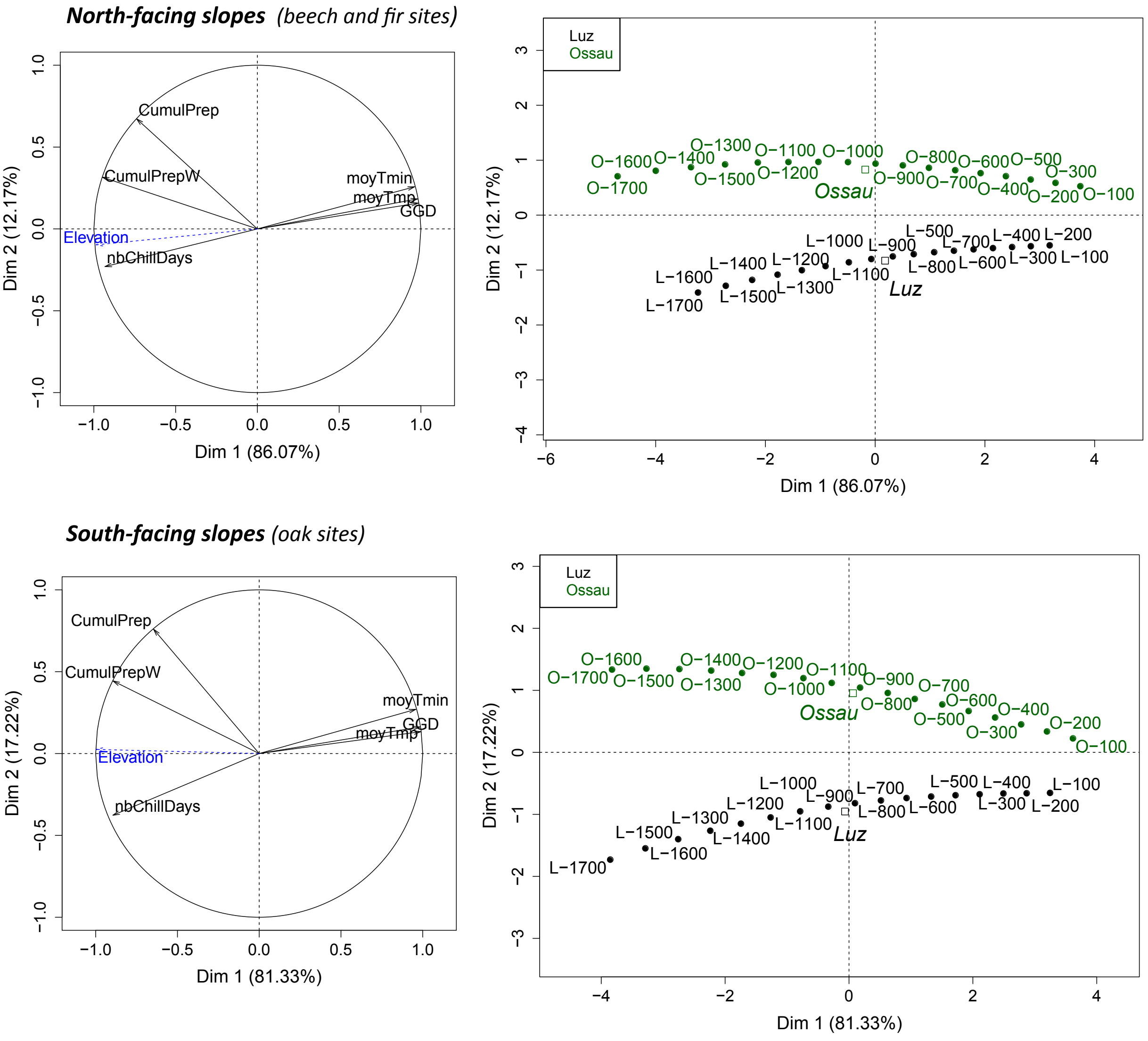

2. The PHENOFIT model

The PHENOFIT model is a process-based tree species distribution model (developed by Chuine and Beaubien 2001). The version of the model used in this study has already been presented in the Supplementary Information of two former studies (Gritti *et al.* 2013, Duputié *et al.* 2015). We reported here the most relevant parts of these previously published descriptions of PHENOFIT, and some additional information concerning the model calibration chosen for the present study case. We used the model calibrated for beech and oak by Duputié *et al.* (2015), and we fully calibrated the model for fir. For all species, we modified the calibration of the budburst sub-model included in PHENOFIT so that the model simulates as best as possible the phenology of the reference populations.

The model PHENOFIT relies on the assumption that species adaptation to abiotic conditions is tightly related to its capacity to synchronize its annual life cycle with seasonal climatic variations directly impacting its survival and reproductive success. It simulates the precise phenology and levels of resistance to drought and cold stress of an average individual of a tree species given local climatic conditions to yield a reproductive success and a survival probability. All sub-models describing how the different functional traits vary with the environmental conditions, and how functional traits determine annual survival and reproductive success, have been formalized using our current understanding of the eco-physiology of the species and extensive experimental and empirical work. The product of survival and reproductive success is often used as a proxy for fitness, and for the probability of occurrence. Note that the observed species' distribution is not used to calibrate the model parameters..

a. Phenology

Budburst and flowering dates

Budburst and flowering dates were determined by daily temperatures using the Unichill model which is a sequential two-phases model describing the cumulative effect of chilling temperatures on bud development during the endodormancy phase (first phase) and the cumulative effect of forcing temperatures during the ecodormancy phase (second phase). The end of the endodormancy phase, t_c, occurs after the accumulated rate of chilling, R_c, reaches a critical sum of chilling units, C^*:

$$\sum_{t_0}^{t_c} R_c(T_d) \geq C^* \tag{4}$$

with t_0 the beginning of the endodormancy phase.

Budburst occurs at date t_f, when the accumulated rate of forcing, R_f, reaches a critical amount of forcing units, F^*:

$$\sum_{t_c}^{t_f} R_f(T_d) \geq F^* \tag{5}$$

Following Gauzere *et al.* (2017), we chose to define the response to chilling temperatures as a threshold function for beech and oak:

$$R_c(T_d) = \begin{cases} 1 \text{ if } T_d < T_b \\ 0 \text{ if } T_d \geq T_b \end{cases} \quad (6)$$

with T_d, the mean temperature of day d and T_b, the threshold temperature (also called base temperature) of the function.

For fir, the response to chilling temperatures was best modelled by the following reaction norm (Chuine 2000):

$$R_c(T_d) = \frac{1}{1 + e^{[a(T_d - c)^2 + b(T_d - c)]}} \quad (7)$$

For all species, we defined the response to forcing temperatures (T_d), also called rate of forcing R_f, as a sigmoid function:

$$R_f(T_d) = \frac{1}{1 + e^{-d_T(T_d - T_{50})}} \quad (8)$$

with d_T the positive slope and T_{50} the mid-response temperature of the sigmoid function. Since beech and oak have mixed buds containing both leaves and flowers, flowering date was calculated with the same model as budburst, considering that $F^*_{flowering} = F^* + \Delta F$. We did the same for fir, although vegetative and reproductive buds are different, because we had not enough data on flowering date to fully calibrate the Unichill model.

Fruit maturation date

Fruit maturation date was simulated following Chuine and Beaubien (2001), using a sequential model that first describes the effect of warm temperatures on fruit cell multiplication (R_{f_m}) and then the accumulation of assimilates that depends on the proportion of leaves uninjured by frost, warm temperatures (f_W) and water availability.

The response to forcing temperature during the first phase of fruit cell multiplication is modelled with a sigmoid function as:

$$R_{f_m}(T_d) = \frac{1}{1 + e^{-aa(T_d - bb)}} \quad (9)$$

The end of the first phase (t_{fr}) occurs when $\sum_{t_f}^{t_{fr}} R_{f_m}(T_d) \geq F^*$.

The response to warm temperatures during the accumulation of assimilates (second phase) is then defined as:

$$f_W(T_d) = Max[(2(T_d - T_{min})^{\alpha}(T_{opt} - T_{min})^{\alpha} - \frac{(T_d - T_{min})^{2\alpha}}{(T_{opt} - T_{min})^{2\alpha}}, 0] \quad (10)$$

with $\alpha = ln(2)/ln(\frac{T_{max} - T_{min}}{T_{opt} - T_{min}})$, and T_{min} = -5, T_{max} = 40. The fruit maturation date occurs when the accumulation of assimilates reaches *matmoy*.

Leaf senescence date

For the deciduous species, leaf senescence is modelled as a decreasing function of photoperiod and temperatures (following Delpierre et al. 2009). More particularly, once the photoperiod has

decreased below P_b, *i.e.* $P_d < P_b$, the photothermal units accumulates as:

$$R_{sen}(d) = \begin{cases} [T_b - T_d]^{\alpha} \times \left(\frac{P_d}{P_b}\right)^{\beta} \text{ if } T_d < T_b \\ 0 \text{ if } T_d \geq T_b \end{cases} \tag{11}$$

with T_d and P_d the temperature and the photoperiod of the day d. The parameters α and β take discrete values to describe the effects of temperature and photoperiod to be included. The date of leaf senescence occurs when: $\sum_t^{ts} R_{sen}(d) \geq S^*$.

b. Survival to stresses

Two kinds of stress are considered: frost and drought. A lethal frost temperature is used in the model but actually never plays a role in determining species range limits in historical climatic conditions. Frost injury on buds, leaves, flowers and fruits is modelled according to the model of Leinonen (1996). Frost hardiness depends upon the organs' developmental stage, photoperiod and temperature. Frost hardiness is highest during the dormancy phase, and lowest during budburst. Frost can injure buds, leaves, flowers and fruits. The minimal and maximal frost hardiness parameter of the different organs ($FHmin$, $FHmax$), and the parameters of the responses to temperature (Te) and photoperiod (NL) of frost hardiness are detailled in Table S2.
In this version of PHENOFIT, survival to drought or flooding was implemented as follows: four limits of yearly precipitation were set; outside the outer precipitation limits, survival was assumed to be 0.1; inside the inner limits, it was assumed to be 1 and it varied linearly between the inner and outer limits.

c. Reproductive success

The reproductive output corresponds to the proportion of mature fruits produced by the end of the year. It is calculated as the product of fruit maturation success and the proportion of fruits that reach maturation (*i.e.* not killed by frost all along the season since the flower primordia). The number of flowers initiated each year per tree was not simulated here, as it relies on other traits and processes than phenology (*e.g.* nutrient allocation, tree size and age, architecture). The proportion of fruits that reach maturity undamaged by frost is calculated following the frost damage model of Leinonen (1996) parametrized for flowers and fruits. The success of maturation depends upon the proportion of uninjured leaves available for photosynthesis following a sigmoid function with parameter $pfe50$, the proportion of injured leaves that reduces by 50 % the flux of photosynthetic assimilates going to the fruits. It also depends on a drought index calculated with a daily water balance using precipitation, actual evapotranspiration and soil water holding capacity. Finally, it depends on temperature which determines the course of maturation. Fruits maturation date follows a normal distribution within the tree crown, defined by $E_c \sim \mathcal{N}(matmoy, sigma)$, with $matmoy$ and $sigma$ expressed as a sum of developmental units and sigma chosen so that 95 % of the fruits mature within one month (Chuine and Beaubien 2001).

d. Input and output variables

PHENOFIT input variables are daily climatic data: minimum and maximum temperature, relative humidity, rainfall, global radiation, wind speed. Given the climate and species-specific reaction norms, PHENOFIT then simulates the occurrence date of key phenological events and

the abilities of leaves, flowers and fruits to resist temperature and water stresses. The model outputs the yearly phenological dates (DOY) as well as a yearly survival and reproductive success, within [0;1]. The fitness proxy we used in our analyses is the predicted average reproductive success of an adult tree over the 1960-2012 period. Note that this reproductive success is a relative measure (ranging from 0 to 1), and that the variation in the number of initiated flowers (and subsequent number of fruits) is not simulated by the model.

e. Model calibration

Phenology models were calibrated using the PMP free software (https://www.cefe.cnrs.fr/fr/ressources/logiciels?id=921) and phenological observations, as well as corresponding meteorological data. Calibration was performed on about 65 % of the data (randomly subset) so that an external validation of the models could also be done. Each phenology sub-model was calibrated by minimizing the residual sum of square using the simulated annealing algorithm of Metropolis (Chuine *et al.* 1998). The performance of each model was then evaluated based on three indices:

$$EFF = 1 - \frac{\sum_{i=1}^{n}(O_i - P_i)^2}{\sum_{i=1}^{n}(O_i - \bar{O})^2} \; ; \qquad \text{with } EFF \in [-\infty; 1]$$

$$RMSE \text{ or } RMSEP = \sqrt{\frac{\sum_{i=1}^{n}(O_i - P_i)^2}{n}}$$

$$\theta = \frac{\sum_{i=1}^{n}(O_i - P_i)}{n}$$

with O_i the observation at site i, $\bar{O}$ the average observation value, P_i the prediction at site i, n the number of observations, k the number of parameters, $SStot$ the total sum of square and $SSres$ the residual sum of squares of a given model.
In addition to statistical performances, we evaluated the relevance of the models based on the biological realism of the adjusted reaction norms (Gauzere *et al.* 2017).

For oak, beech and fir, we calibrated the budburst sub-model using phenological observations from the reference populations in the Pyrenees, and other sites in France. The robustness of the model was ensured by the fact that we used a lot of phenology observations (up to 175 observed dates) monitored in a large range of environmental conditions (see Table S3). The overall model accuracy to predict budburst dates was higher for oak and beech ($RMSE$ = 6.98 and 7.38 respectively) than for fir ($RMSE$ = 10.23; Table S3).
For oak and beech, we used the calibration performed by Duputié *et al.* (2015) for the other phenology sub-models. For fir, for which a complete calibration of PHENOFIT had never been performed, we also calibrated the other phenology sub-models using observations for French populations (see Table S2). Models of frost and drought resistance were parameterized using parameter values from the literature (Sakai and Larcher *Ecological Studies*; Lenz *et al.* 2013). The parameters adjusted for the species-specific models, and the origin of all parameter values, are provided in Table S2.

f. Model simulations

Simulations were performed using daily climatic variables generated from 1959 to 2012 for 34 sites ranging from 100 to 1700 m in south-facing slopes for beech, 34 sites ranging from 100 to 1700 m in north-facing slopes for oak and 20 sites ranging from 800 to 1700 m in south-facing slopes for fir. We used a water holding capacity of 150 mm and the Penman-FAO model for

evapotranspiration. Simulations were also performed with a water holding capacity of 60 mm with no qualitative change in the results.

g. Model validation

The validation of the PHENOFIT model was performed using different type of data and at different spatial scales. The main way PHENOFIT was validated so far relied on the comparison between the observed presence/absence data at the scale of the species range and the modelled variation in fitness at this scale, by calculating an AUC index ("area under the receiver operating characteristic curve"). The main advantage of the AUC index ([0; 1]) is that it estimates the level of agreement between observed presence and predicted fitness (AUC of 1 indicating perfect agreement), without having to define a fitness threshold above/under which the species can/cannot be present. Duputié *et al.* (2015) performed this validation for oak and beech, and showed that the modelled fitness matched with a very good accuracy the known distribution of these trees at the European scale (AUC = 0.84 for beech, AUC = 0.87 for oak; Fig. S3a). For fir, this quantitative comparison was performed at the scale of France, and PHENOFIT also showed a good accuracy to model the species range (AUC = 0.72; Fig. S3b).
We also validated the PHENOFIT model at the scale of the reference sites in the Pyrenees. We first compared the observed budburst dates, measured from 2005 to 2012, to the predicted dates using the specific temperature data for these sites (measured by Hobbo sensors, Part S1). For the three species, the model simulated with a very good accuracy the spatio-temporal variation in budburst dates, and captures between 74 % and 99 % of the spatial variation depending on the species (Fig. S4).
We also compared the observed and predicted variation in fitness components along the elevation gradient. For all species, PHENOFIT predicted that the spatial variation in fitness was mostly due to variation in reproductive success and not survival, which varied very little (Fig. S5). Focusing only on this reproductive performance index, we compared the predicted spatial variation in fitness with the mass of acorns and height of trees measured in the references sites for oak and beech (Fig. S6). Note that these two different measures of tree performance (fecundity and growth) are expected to be constrained by the length of the growing season, which is affected by the budburst date and the local climate. Even though the number of observed data points is too limited to allow a quantitative evaluation of the performance of the model, it can qualitatively be compared to our simulations. For oak, we predicted a sharp decline in reproductive success between 500 and 1000 m, and null fecundity after 1200 m, which s consistent with the observed variation in fecundity in the reference sites (Fig. S6a). For beech, we predicted a sharp decline in reproductive success after 1300 m. For this species, for which we do not have fecundity data, this prediction is consistent with the observed variation in height growth showing an optimum around 500 m, and decreasing after 1000 m (Fig. S6b). However, predicted fitness for beech at the lowest elevation did not match the low height of this population, possibly due to an underestimation of drought at this site (Fig. S6b). For fir, for which we predicted almost no spatial variation in fitness, we do not have fecundity or growth data to compare to the model prediction.
It is however important to note that, in all cases, these empirical data are not strictly comparable to the PHENOFIT output (*i.e.* average reproductive success over the 1960-2012 period), as they were measured on young trees for height growth and over a few successive years for fecundity. Yet, these observations still represent the best data available to perform a cross-validation of our models at a local scale, and they show that, qualitatively, our predictions are biologically

relevant and reproduce patterns observed in the field.

h. Local sensitivity analysis

A local sensitivity analysis of the PHENOFIT model was performed to investigate the relationship between budburst date (z) and fitness (w). To that purpose, we first simulated different budburst dates in a biologically credible range by varying a parameter of the budburst model ($T_{50} \in$ [-20; 30]), the other parameters of PHENOFIT remaining set to the value adjusted for each species (Table S2). The credible range was determined using budburst date observations at the scale of the European distribution of the species (PEP725 database http://www.pep725.eu/TEMPOdatabasehttps://www6.inra.fr/soere-tempo and data from the Pyrenean study sites): $z \in [60; 170]$ for beech and oak and $z \in [100; 220]$ for fir. The chosen T_{50} parameter corresponds to the mid-response temperature of the cell elongation during ecodormancy (eq. 8). It has been shown to have the largest effect on the predicted budburst dates as compared to other physiological parameters (Gauzere *et al.* 2019). For each model run and simulated budburst date, PHENOFIT predicted the resulting fitness. The local sensitivity analysis thus allowed characterizing the individual fitness functions ($w(z)$), which is the relationship between the reproductive success (w) and the budburst date (z).

Table S2. Parameter values of the PHENOFIT model used for each species. The origin of the data used to adjust each sub-model, or the reference to previously published parameter values, are indicated in parentheses. n_{obs} provides the number of data used to calibrate each phenology submodel.

	F. sylvatica	***Q. petraea***	***A. alba***
Budburst date	(https://oreme.orgobservation/ods/) n_{obs} = 97	(https://oreme.orgobservation/ods/, https://quercusportal.pierroton.inra.fr/) n_{obs} = 175	(https://oreme.orgobservation/ods/, https://quercusportal.pierroton.inra.fr/) n_{obs} = 78
t_0	-62.0	-63	-67
C^*	73.7	116.7	144.6
F^*	37.3	23.0	9.0
	T_b 13	T_b 13.4	a 1.61
	-	-	b -27.1
	-	-	c -4.95
d_T	0.08	0.41	0.24
T_{50}	13.0	9.0	15.2
Flowering date	(https://oreme.orgobservation/ods/) n_{obs} = (97 +) 21	(https://oreme.orgobservation/ods/) n_{obs} = (175 +) 92	(https://oreme.orgobservation/ods/) n_{obs} = (78 +) 1
t_0	-62.0	-63	-67
C^*	73.7	116.7	144.6
F^*	40.3	29.0	16.0
	T_b 13	T_b 13.4	a 1.61
	-	-	b -27.1
	-	-	c -4.95
d_T	0.08	0.41	0.24
T_{50}	13.0	9.0	15.2
Fruit maturation date	(https://oreme.orgobservation/ods/) n_{obs} = 23	(https://oreme.orgobservation/ods/) n_{obs} = 296	(https://oreme.orgobservation/ods/) n_{obs} = 33
aa	-0.2	0.67	23.8
bb	14.7	10.3	7.8
F^*	9.0	87.1	35.8
T_{opt}	5.0	5.0	0.28
$matmoy$	100.0	66.3	51.1
$sigma$	3.7	4.59	2.44
$pfe50$	0.6	0.6	0.9
$photosType$	2	2	2

Table S2. following

	F. sylvatica	*Q. petraea*	*A. alba*
Leaf senescence date	(https://oreme.orgobservation/ods/) n_{obs} = 560	(https://oreme.orgobservation/ods/) n_{obs} = 228	(https://oreme.orgobservation/ods/) -
P_b	13.4	11.3	-
T_b	29.9	22.2	-
α	1.0	2.0	-
β	2.0	0.0	-
S^*	570.6	1340	-
Frost hardiness	(Leinonen *Ann. Bot.* 1996, Lenz et al. *New Phytol.* 2013)	(Leinonen *Ann. Bot.* 1996, Lenz et al. *New Phytol.* 2013)	(Leinonen *Ann. Bot.* 1996, Sakai and Larcher *Ecological Studies* 62)
$FHfrmax1$	-6.0	-12.0	-12.0
$FHfrmax2$	-20.0	-50.0	-18.0
$FHminfe$	-5.3	-5.4	-4.0
$Te1$	10.0	10.0	10.0
$Te2$	-16.0	-16.0	-16.0
$FHtfemax$	-8.0	-10.0	-16.0
$FHpfemax$	-12.0	-14.0	-9.0
$NL1$	10.0	10.0	10.0
$NL2$	16.0	16.0	16.0
$FHminfl$	-5.3	-4.4	-4.0
Survival to drought	(https://inventaire-forestier.ign.fr/)	(https://inventaire-forestier.ign.fr/)	(https://inventaire-forestier.ign.fr/)
$ppextremelow$	450	460	600
$pplow$	490	490	800
$pphigh$	3000	3000	3000
$ppextremehigh$	3500	3500	3500

Table S3. Performance of the budburst model UniChill with the calibration dataset and external validation dataset for the three studied species. Here, phenology observations from the reference populations in the Pyrenees and other sites in France were used to improve the efficiency of the budburst sub-model.

	F. sylvatica	*Q. petraea*	*A. alba*
Calibration performance			
n	97	175	78
k	6	6	8
$SStot$	8589.9	32918.4	10960.4
$SSres$	5284.0	8530.4	8163.9
$RMSE$	7.38	6.98	10.23
EFF	0.39	0.74	0.26
Validation performance			
n	39	101	23
$RMSE_P$	7.75	7.36	13.3
θ	-0.34	-1.73	5.03

Figure S3. Global-scale validation of the PHENOFIT model for beech, oak and fir. The observed species distribution range (presence/absence data) at the European (**a**) or French (**b**) scale is compared to the fitness predicted by the model. AUC index ([0; 1]) were calculated to estimate the level of agreement between observed presence and predicted fitness over Europe or France (AUC of 1 indicating perfect agreement). For beech and oak, this validation was previously performed by Duputié *et al.* (2015). Presence/absence maps are consensual maps based on the compilation of different data sources (see Duputié *et al.* 2015 for further details). For fir, the fitness threshold of 0.09 was chosen to predict its range (Leys and Chuine, *pers. comm.*).

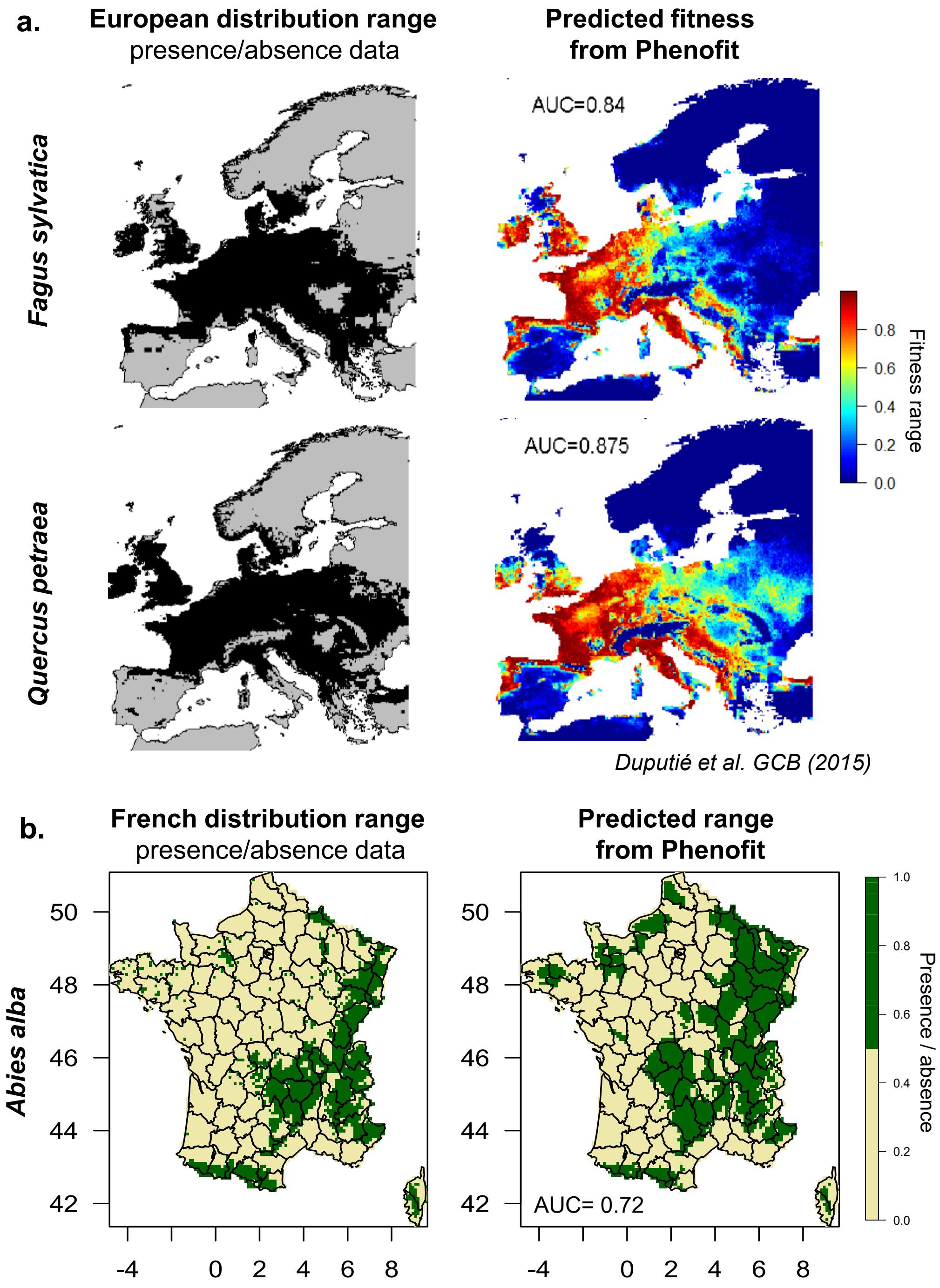

Figure S4. ***a.*** Performance of the phenology model to predict the spatio-temporal variation in budburst dates observed in the reference populations. The R-squared of the linear regression between predicted and observed budburst dates is of $R^2 = 0.88$ for oak, 0.69 for beech and 0.82 for fir (dotted lines represent the 1:1). ***b.*** Average budburst dates observed *in situ* (dots) and predicted by the model (crosses) along the Pyrenean elevation gradients. The error bars provide the standard deviation due to the yearly fluctuation of phenology. The budburst model predicts with a good to very good accuracy the observed spatial variation of phenology dates, with $R^2 = 0.92$ for oak, 0.74 for beech and 0.99 for fir. Note that the lower performance of the model in beech is due to the particularly early budburst phenology at elevation 1260 m, probably due to the micro-climate of this population. If we remove this population then $R^2 = 0.87$.

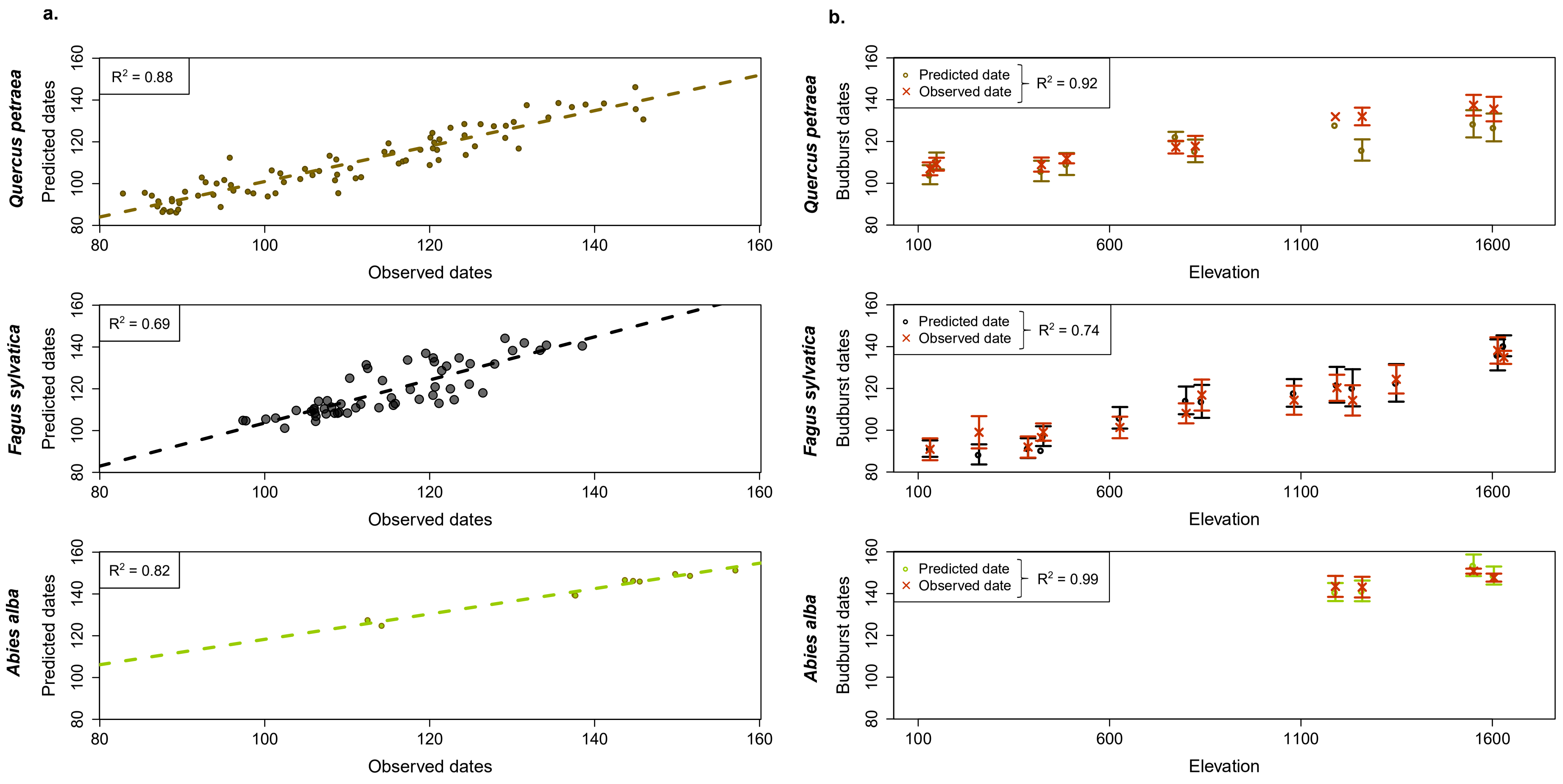

Figure S5. Spatial variation in (a.) reproductive success and (b.) survival predicted by the PHENOFIT model for the three studied species (average estimates for the period 1960-2012). The simulations were performed using the parameters adjusted for each species (Table S2) and the simulated climate along the Gave valley (Part S1). As expected for long-lived species such as trees, fitness variation along climatic gradients is mostly due to variation in reproductive success, and to a minor extent to variation in survival.

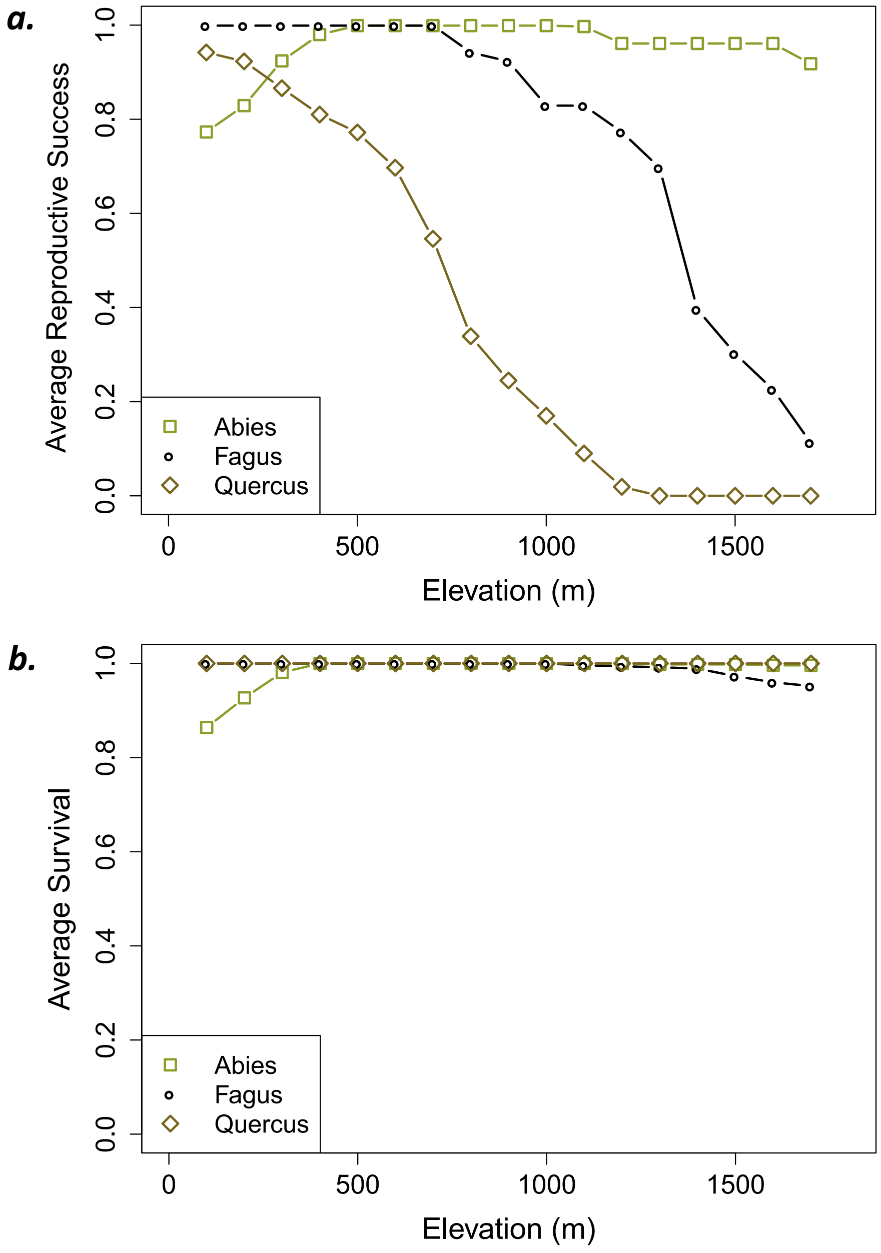

Figure S6. Comparison of observed and predicted performance of oak (a) and beech (b) along the elevation gradient in the Gave valley. Observed height corresponds to height of 11-year old oak and beech trees in a reciprocal transplant experiment located at 5 elevations along the Gave valley (see Vitasse *et al.* 2010). Observed acorn production for oak corresponds to the mean production of adult trees of the reference populations over 6 years. Although not strictly comparable to the PHENOFIT output (*i.e.* average reproductive success over the simulated 1960-2012 period), these data are the only ones available to evaluate the model performance at this very local scale.

a. *Quercus petraea*

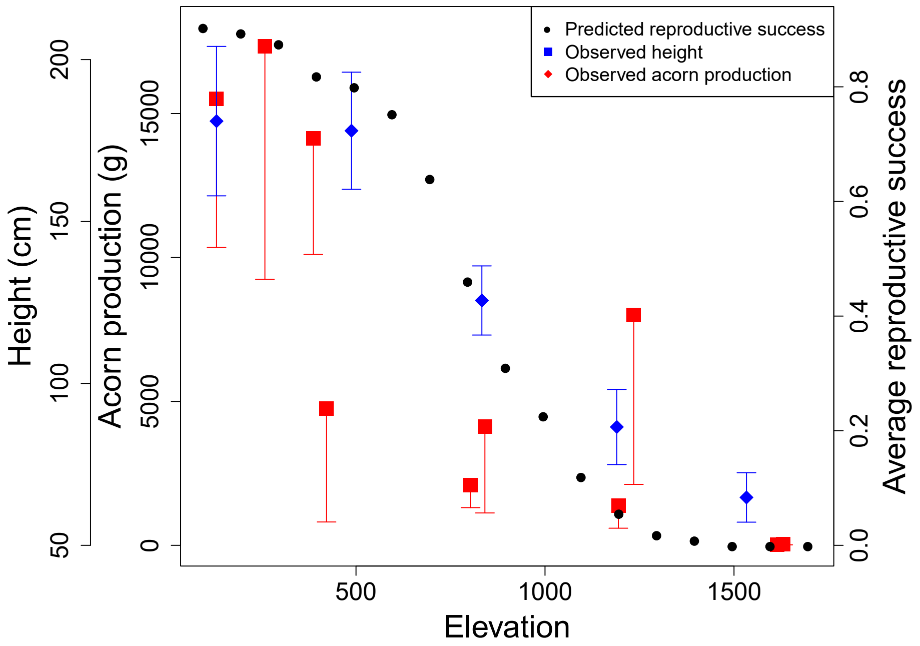

b. *Fagus sylvatica*

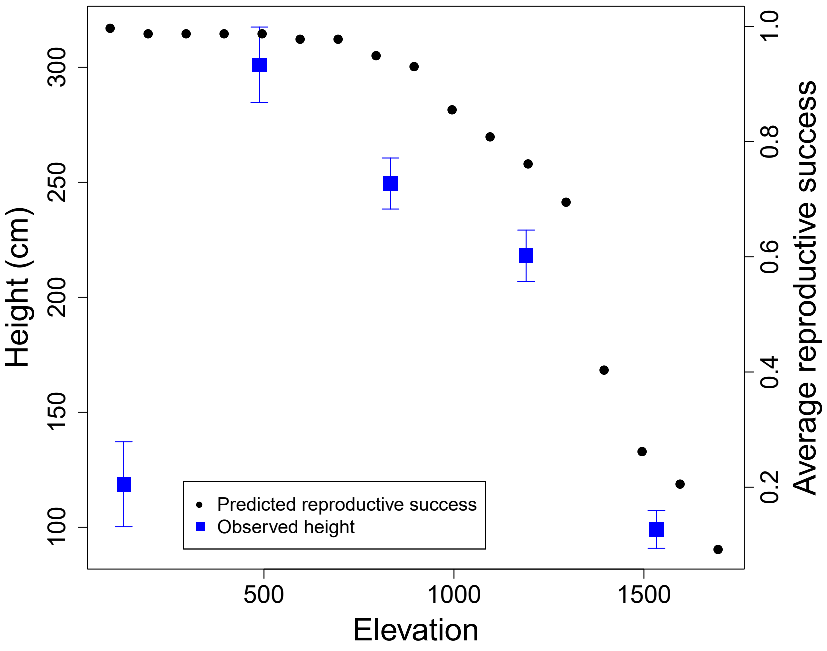

Supplement: Supplementary file 1 — Table S1. Coefficient values for the equations used to simulate the elevation gradients for each valley and species. Table S2. Parameter values of the PHENOFIT model used for each species. Table S3. Statistical performance of the budburst model. Figure S1. Location and reference of the SAFRAN reference points. Figure S2. Climatic characterization of the simulated elevational gradients. Figure S3. Global‐scale validation of the PHENOFIT model using observed species distribution range and AUC. Figure S4. Spatio‐temporal variation in observed and predicted budburst dates for the reference sites in the Pyrenees. Figure S5. Reproductive success and survival predicted by the PHENOFIT model across the reference sites in the Pyrenees. Figure S6. Spatial variation in predicted fitness and observed performance of beech and oak trees in the Pyrenees (Gave valley). Data S1. [file EVL3-4-109-s001.pdf]
